# The Transcription Factor Prdm16 Marks a Single Retinal Ganglion Cell Subtype in the Mouse Retina

**DOI:** 10.1167/iovs.17-22442

**Published:** 2017-10

**Authors:** Sergio Groman-Lupa, Joseph Adewumi, Ko Uoon Park, Joseph A. Brzezinski

**Affiliations:** Department of Ophthalmology, University of Colorado Denver, Aurora, Colorado, United States

**Keywords:** retina, retinal ganglion cell, Prdm16, vasculature, development, cell fate, ganglion cell subtype

## Abstract

**Purpose:**

Retinal ganglion cells (RGC) can be categorized into roughly 30 distinct subtypes. How these subtypes develop is poorly understood, in part because few unique subtype markers have been characterized. We tested whether the Prdm16 transcription factor is expressed by RGCs as a class or within particular ganglion cell subtypes.

**Methods:**

Embryonic and mature retinal sections and flatmount preparations were examined by immunohistochemistry for Prdm16 and several other cell type-specific markers. To visualize the morphology of Prdm16+ cells, we utilized *Thy1-YFP-H* transgenic mice, where a small random population of RGCs expresses yellow fluorescent protein (YFP) throughout the cytoplasm.

**Results:**

Prdm16 was expressed in the retina starting late in embryogenesis. Prdm16+ cells coexpressed the RGC marker Brn3a. These cells were arranged in an evenly spaced pattern and accounted for 2% of all ganglion cells. Prdm16+ cells coexpressed parvalbumin, but not calretinin, melanopsin, Smi32, or CART. This combination of marker expression and morphology data from *Thy1-YFP-H* mice suggested that the Prdm16+ cells represented a single ganglion cell subtype. Prdm16 also marked vascular endothelial cells and mural cells of retinal arterioles.

**Conclusions:**

A single subtype of ganglion cell appears to be uniquely marked by Prdm16 expression. While the precise identity of these ganglion cells is unclear, they most resemble the G_9_ subtype described by Völgyi and colleagues in 2009. Future studies are needed to determine the function of these ganglion cells and whether *Prdm16* regulates their development.

A diverse set of specialized cell types are required for normal vision. How this cellular diversity is achieved during development is only partially understood. The vertebrate retina provides an ideal system to address this question because it has a large number of discrete cell types based on morphology, physiology, and gene expression patterns. Retinal ganglion cells (RGCs) are the sole output neurons of the retina, relaying photic information through their axons to several areas of the brain.^1^ Not only are ganglion cells essential for visual perception, they are also needed for circadian photoentrainment and ocular reflex behaviors.^2,3^

In the mouse, RGCs are formed from embryonic day (E) 11.5 to around birth, with peak production near E13.5.^4–7^ Experiments have identified several transcription factors that control ganglion cell development. *Atoh7* (*Math5*) is required for retinal precursors to acquire the potential to adopt ganglion cell fate.^8–10^ Animals lacking *Atoh7* do not generate RGCs.^11–13^ Downstream of Atoh7 are the Brn3 transcription factors. Brn3a (Pou4f1); Brn3b (Pou4f2); and Brn3c (Pou4f3) are made only by ganglion cells in the retina.^14^ *Brn3b* mutants lack most ganglion cells and mutations in all three genes cause dendritic and axon pathfinding defects.^15–23^ Loss of other transcription factors expressed by developing RGCs, such as *Isl1*, *Tbr2*, *Sox4*, and *Sox11*, reduces the number of ganglion cells.^24–28^ Conversely, simultaneous overexpression of *Brn3b* and *Isl1* is sufficient to generate RGCs.^29^

Like most retinal neuronal classes, ganglion cells can be further divided into several subtypes. In the mouse, more than 30 ganglion cell subtypes are predicted based on morphology, physiology, and marker expression.^1,30–37^ These neurons have been loosely categorized based on whether they fire upon the onset (ON) or loss (OFF) of light stimuli. This correlates with the location of ganglion cell dendrite stratification in the inner plexiform layer (IPL). ON dendrites localize to the inner half of the IPL and OFF dendrites localize to the outer half. Some ganglion cells are bistratified, with dendrites in both the ON and OFF layers of the IPL. While each ganglion cell subtype is thought to have a unique gene expression profile, few have been uniquely identified with markers or transgenic reporter mice to date (for review, see Ref. 32).^32,38–40^ Although much has been learned about RGC development as a class of neurons, little is known about how individual subtype identities are acquired. Transcription factors are predicted to regulate ganglion cell subtype fate choice; however, they tend to mark multiple ganglion cell subtypes. For example, the three Brn3 transcription factors each mark several subsets of ganglion cells in an overlapping fashion.^19^ These data suggest that the rare intersection of more widely expressed transcription factors regulates ganglion cell subtype identity.^23,32,39^ An alternative model is that subtype identity is regulated by uniquely expressed transcription factors.

Recently, several groups have investigated the expression and function of the *Prdm* family of transcription factors in the retina. The 16 *Prdm* genes in the mouse are characterized by a PR-SET methyltransferase domain and multiple C_2_H_2_ zinc-finger motifs.^41,42^ *Prdm1*, *Prdm8*, and *Prdm13* have been best characterized in the retina, where they have each been shown to regulate cell fate choice.^43–51^ We examined the mRNA expression of the remaining 13 *Prdm* genes by RT-PCR and RNA-seq and found that most of them are expressed during development, including *Prdm16*.^52^ Prdm16 has been characterized in several developmental systems. It controls the development of chondrocytes, a subset of neural progenitors in the brain, brown adipose fate choice, and is required for hematopoietic stem cell maintenance.^41,53–60^ Within the eye, Prdm16 was shown to be expressed by the retinal pigmented epithelium (RPE).^57,61^

Here, we have examined the expression of Prdm16 throughout murine retinal development by immunohistochemistry. As previously reported,^57^ we observed nuclear Prdm16 staining of the RPE at all ages examined. While generally absent from the retina, we found that Prdm16 marked 2% of ganglion cells starting late in embryonic development. Using a series of molecular markers, Prdm16 appeared to mark a single ganglion cell subtype that is similar to the G_9_ subtype described by Völgyi and colleagues.^30^ This raises the possibility that unique subtype-specific transcription factors regulate RGC subtype fate choice during retinal development. We also observed that a subset of large diameter blood vessels expressed Prdm16, both inside and outside the eye. In the retina, these vessels were always arterioles. Prdm16 marked the two major vascular cell types, endothelial and mural cells. This pattern of expression suggests that Prdm16 controls arterial vessel development and/or function, both in the retina and more broadly throughout the animal.

## Methods

### Animals

All mice were used in accordance with the ARVO Statement for the Use of Animals in Ophthalmic and Vision Research and with the approval of the University of Colorado Denver Institutional Animal Care and Use Committee. Wild-type CD-1 mice were used for retinal sections at embryonic and postnatal stages. *Thy1-YFP-H* mice were acquired from Jackson Laboratories (stock #3782; Bar Harbor, ME, USA)^62^ and maintained by outcrossing to CD-1 animals (Charles River Laboratories, Wilmington, MA, USA). Flatmount stains were done with CD-1 mice or the wild-type littermates of *Thy1-YFP-H* mice. The Prdm16 staining pattern in retinal flatmounts (below) was equivalent in *C57BL/6J* mice (Jackson Laboratories, stock #664) and at all ages examined (3–25 weeks; data not shown).

### Histology

The heads of embryos were fixed for 2 hours in 2% paraformaldehyde and cryopreserved through 30% sucrose and frozen in optimal cutting temperature (OCT; Sakura, Torrance, CA, USA). For postnatal retinas, eyes were fixed for 15 minutes in 2% paraformaldehyde and the cornea and lens removed. The eyes were fixed for an additional 75 minutes in 2% paraformaldehyde, cryopreserved, and frozen in OCT. Horizontal sections were cut at 10 μm and immunostained as previously described.^43,52^ For adult retinal flatmounts, eyes were fixed and the cornea and lens removed, as above. These eye cups were then blocked for 5 hours with the supernatant of a solution of 5% nonfat milk, 0.5% Triton X-100, in PBS (milk block)^43^ at room temperature. The eye cups were incubated in primary antibodies (1 mL milk block per eye) for 36 to 65 hours at 4°C, washed with PBS + 0.1% Triton X-100, and incubated with AlexaFluor conjugated secondary antibodies or streptavidin (1 mL milk block per eye; Jackson Immunoresearch, West Grove, PA, USA) for 4 to 6 hours at room temperature. The eye cups were washed as above. The retinas were dissected from the eye cups, cut radially, and then flattened onto microscope slides for imaging. When *Gsi*B4 lectin was used, the milk block was replaced with 10% normal donkey serum, 1% BSA, 0.5% Triton X-100 in PBS to prevent lectin binding to the sugars in the milk. The solutions for primary and secondary steps were the same except that they contained 3% normal donkey serum. The following primary reagents were used: mouse anti-Ap2α (1:250, 5E4-c; Developmental Hybridoma Studies Bank [DHSB], Iowa City, IA, USA); mouse anti-Brn3a (1:250, MAB-1585; Millipore Corp., Billerica, MA, USA); mouse anti-calretinin (1:500, MAB-1568; Millipore Corp); rabbit anti-cocaine and amphetamine regulated transcript (CART, 1:1000, H-003-62; Phoenix Pharmaceuticals, Burlingame, CA, USA); rabbit anti-Erg1 (1:250, ab92513; Abcam, Cambridge, MA, USA); chicken anti-GFP (1:1000, ab13970; Abcam); *Griffonia simplicifolia* isolectin B4-biotin (*Gsi*B4, 1:250, B-1205; Vector Laboratories, Burlingame, CA, USA); mouse anti-Isl1/2 (1:100, 39.4D5-c, DHSB); rabbit anti-melanopsin (1:1000, gift from Russel Van Gelder, University of Washington)^63^; mouse anti-non-phosphorylated neurofilament H (Smi32, 1:500, Smi-32R; Covance, Princeton, NJ, USA); mouse anti-parvalbumin (1:250, PV25; Swant, Marly, Switzerland); rat anti-Pdgfrβ (1:250, NBP1-43349; Novus Biologicals, Littleton, CO, USA); goat anti-Prdm16 (1:20, sc55697; Santa Cruz Biotechnology, Dallas, TX, USA); sheep anti-Prdm16 (1 μg/mL, AF6295; R&D Systems, Minneapolis, MN, USA); rabbit anti-α-smooth muscle actin (αSMA, 1:100, ab5694; Abcam); and rabbit anti-Tbr2 (1:500, ab23345; Abcam). The sheep anti-Prdm16 antibody (R&D Systems) was shown to be specific by comparing brown adipocytes from wild-type and *Prdm16* conditional knockouts.^64^ Images were acquired with laser scanning confocal microscopes (Olympus FV1000; Center Valley, PA, USA, and Nikon C2; Melville, NY, USA). Z-stack projections or orthogonal views were generated in ImageJ (http://imagej.nih.gov/ij/; provided in the public domain by the National Institutes of Health, Bethesda, MD, USA).^65^ Images were minimally processed with a raster graphics editor (Photoshop; Adobe Systems, San Jose, CA, USA).

### Quantification and Spatial Statistics

We immunostained 15 retinas from 15 wild-type mice with Brn3a, parvalbumin, and Prdm16 and imaged one representative ×200 magnification field from each for quantification. This represented 485 Prdm16+ cells. The total number of Brn3a+ cells, Prdm16+/Brn3a+, and Prdm16+/parvalbumin+ cells was counted and averaged. The maximum parvalbumin+ soma diameter was measured using ImageJ^65^ for six of these fields and averaged, representing 129 Prdm16+/parvalbumin+ cells. For the seven Thy1-YFP+/Prdm16+ ganglion cells we identified from 107 retinas, the maximum diameter of the soma was measured using ImageJ and averaged. The diameter of the dendritic field for the single Thy1-YFP+/Prdm16+ ganglion that could be reliably imaged was measured with ImageJ. To measure the spatial distribution of Prdm16+ ganglion cells across the retina, we imaged seven adult retinal flatmount preparations (4 mice, 7334 Prdm16+ cells) and divided the retina into dorsal, ventral, nasal, and temporal regions. Prdm16+ ganglion cells were counted from each region and contrasting halves (dorsal versus ventral, nasal versus temporal) compared by unpaired student *t*-tests using spreadsheet software (Excel; Microsoft Corp., Redmond, WA, USA). A value of *P* < 0.01 was considered significant. To measure the distribution from the center of the retina to the periphery, the number of Prdm16+ ganglion cells was quantified from circular areas in a series of ten 250-μm radial steps from the optic nerve. The cumulative distribution of Prdm16+ ganglion cells was plotted and evaluated by linear regression with spreadsheet software (Microsoft Corp.). For the nearest neighbor analysis, we used sixteen ×200 magnification images from 11 eyes (10 mice) immunostained with Prdm16. These 16 fields contained a minimum of 19 Prdm16+ ganglion cells each, for a total of 451 cells. We also measured parvalbumin+ cells in the ganglion cell layer from seven ×200 images (seven retinas, six mice), representing a total of 3182 cells. Using the “Analyze” “Cell Counter” feature in ImageJ, the centers of all Prdm16 nuclei and parvalbumin somas were marked and the image containing only these centers exported and converted to grayscale in a raster graphics editor (Microsoft Corp.). Using the “Spatial Statistics” “Spatial Analysis 2D/3D” plugin^66,67^ for ImageJ, the cumulative distribution of nearest neighbor distances was plotted. We plotted and calculated the G-function spatial distribution index using the following parameters: 10,000 Nb points, 500 samples, 8 μm hardcore, and 5% error. From the plots, we measured the 25th, 50th, and 75th percentile values for each image, converted them into distance units (in μm), and calculated the average. The plugin calculated the G-function spatial distribution index for each image, a test for random distribution.^66,67^ We compared the spatial distribution index between Prdm16+ and parvalbumin+ cells by unpaired student *t*-test and considered a *P* < 0.01 as significant. The SD was calculated based on sampling; including whole retinas, ×200 fields, or individual cell soma diameters.

## Results

### Prdm16 is Expressed by the RPE and a Small Number of RGCs

We and others have characterized the expression of *Prdm* family genes in specific types of retinal neurons.^43–50^ Using RT-PCR and RNA-seq approaches, we determined that most of the sixteen *Prdm* family genes^42^ are expressed in the developing mouse retina.^52^ One of these expressed genes, *Prdm16*, was previously characterized in mouse RPE cells adjacent to the retina.^57^ Since our tissue preparations contain very few RPE cells, we hypothesized that *Prdm16* is expressed by cells that reside within the retina.

**Figure 1:**
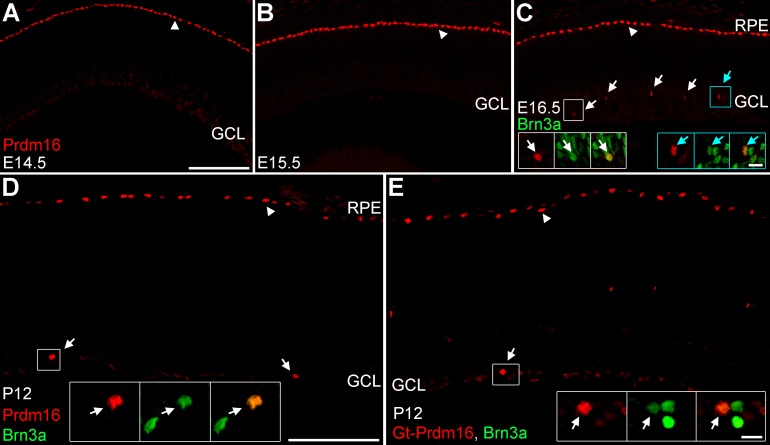
Prdm16 is made by the RPE and some ganglion cells during development. (A–D) Sheep anti-Prdm16 immunostaining (red) of retinal sections. (A, B) At E14.5 (A) and E15.5 (B), Prdm16 nuclear staining is seen in the RPE (arrowheads), but not within the GCL or other areas of the retina. (C) At E16.5, Prdm16 stains the RPE (arrowhead) and some cells in the GCL (arrows). All Prdm16+ cells in the GCL coexpress the ganglion cell marker Brn3a (green) (arrows, insets). (D) At P12, Prdm16 expression marks the RPE (arrowhead) and a small number of Brn3a+ ganglion cells (arrows, inset). (E) Goat anti-Prdm16 immunostaining (red) at P12 marks the RPE (arrowhead) and a subset of Brn3a+ ganglion cells (arrow, insets). The sheep and goat antibodies have equivalent staining patterns. Weak staining is seen in the ganglion cell layer with both antibodies and may represent either spurious signal or low level expression within other cells. Scale bar: 100 μm for (A–C), and 100 μm for (D, E). Scale bars for insets in (C) through (E) are 10 μm.

To determine when and where Prdm16 was expressed, we stained retinal sections at several stages of development with antibodies specific to Prdm16.^64^ In sections of embryonic (E) day 12.5, 14.5, and E15.5 retinas (data not shown and Figs. 1A–B) we observed strong Prdm16 immunostaining of RPE nuclei, as previously described.^57^ No Prdm16+ nuclei were seen in any area of the retina at these ages. Starting at E16.5 we observed a small number of round Prdm16+ nuclei within the retina; specifically in the nascent GCL (Fig. 1C). At this age, the GCL contains amacrine and retinal ganglion cell neurons. To determine whether these two cell types expressed Prdm16, we costained with antibodies against AP2α (amacrine)^68^ and Brn3a (most RGCs).^14^ The Prdm16+ cells in the GCL did not coexpress AP2α (data not shown), rather they all coexpressed Brn3a (Fig. 1C). While RGC-specific at this age, it was unclear whether Prdm16 always marks a small fraction of RGCs or if as development progresses more RGCs become Prdm16-positive. To test this, we examined mouse retinas at postnatal day (P) 12, after the completion of neurogenesis. Prdm16 staining was similar to E16.5, labeling the RPE and a small number of cells in the GCL (Fig. 1D). These Prdm16+ cells always coexpressed Brn3a (Fig. 1D). Thus, Prdm16 marks only a small subset of ganglion cells at both stages. To mitigate the chance of spurious labeling by our sheep anti-Prdm16 antibody, we immunostained retinas with a different Prdm16 antibody raised in goat. The goat anti-Prdm16 antibody labeled the RPE and a small number of Brn3a+ RGCs at P12 (Fig. 1E). This was the same pattern seen with the sheep anti-Prdm16 antibody (Fig. 1D). Equivalent staining with each antibody was seen at other ages (data not shown), strongly suggesting that the RPE and RGC labeling reflects the true Prdm16 expression pattern in the mouse. Since the patterns were equivalent, we used the sheep antibody for the remaining studies as it was the more robust tool.

### Prdm16 Marks a Subset of RGCs

**Figure 2:**
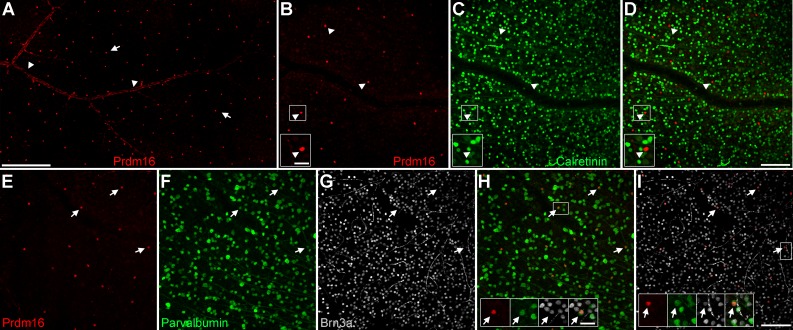
Prdm16+ ganglion cells express Brn3a and parvalbumin. Adult retinal flatmount preparations stained with Prdm16 (red) and ganglion cell markers. (A) Prdm16 immunostaining of the GCL. The image is from midway between the optic nerve (left) and the periphery. Round Prdm16+ nuclei (arrows) are spaced relatively uniformly across the retina. Elongated Prdm16 nuclei (arrowheads) cluster in a pattern reminiscent of blood vessels. (B–D) Prdm16 and calretinin (green) costaining. There are considerably fewer Prdm16+ cells than calretinin-labeled amacrine and ganglion cells. Prdm16+ cells (arrowheads, insets) do not coexpress calretinin. The gap in the staining represents a blood vessel. (E–I) Costaining of Prdm16, parvalbumin (green); and Brn3a (gray). Essentially all round Prdm16+ nuclei coexpress parvalbumin and Brn3a (arrows, insets). The intensity of parvalbumin staining is often modest and the somas are only slightly larger than the Prdm16+/Brn3a+ nuclei (arrows, insets). The bright filamentous staining in the Brn3a images is non-specific labeling of capillaries. Scale bar: 250 μm for (A), 100 μm for (B–D), and 100 μm for (E–I). Scale bars for insets in (B–D) and (H, I) are 25 μm.

There are likely more than 30 distinct ganglion cell subtypes in the rodent retina.^1,30–37^ The sparse labeling we observed suggested that Prdm16 marks a specific ganglion cell subtype. To test this, we examined Prdm16 expression in mature (≥P21) retinal flatmount preparations (Fig. 2). Flatmounts allow for robust quantification of the number and distribution of these rare cells. Round Prdm16+ nuclei were seen throughout the GCL of the retina, forming what appeared to be an evenly spaced array (Fig. 2A). In all retinas, we also observed elongated Prdm16+ nuclei on the inner retinal surface. These nonneuronal nuclei clustered in a pattern reminiscent of large blood vessels (Fig. 2A), described further below.

To narrow the field of possible ganglion cell subtypes, we immunostained retinas with Prdm16 and broadly expressed markers. The first, calretinin, marks most RGCs^69^ (Figs. 2B–D). We observed that Prdm16+ ganglion cells rarely, if ever, coexpressed calretinin (Figs. 2B–D). We next examined parvalbumin, which marks a smaller group of RGCs in the retina^70^ (Figs 2E–I). We observed that essentially all Prdm16+ ganglion cells coexpressed parvalbumin (99.1% ± 1.5% SD, *n* = 15 eyes), though typically at modest levels (Figs 2E–I). The parvalbumin-labeled somas were small (11.14 μm ± 1.06 μm SD, *n* = 129 cells from 6 eyes), only modestly larger than the Prdm16+ nuclei (see insets, Figs. 2H–I). Brn3a marks a large cohort of RGC subtypes.^19^ We stained retinal flatmounts with Brn3a and observed that essentially all Prdm16+ cells coexpressed Brn3a (99.8% ± 0.7% SD, *n* = 15 eyes, 485 Prdm16+ cells; Figs. 2E–I). This represented 2.8% (±0.3% SD) of the Brn3a ganglion cells in the retina (Figs. 2E–I). Since Brn3a marks about 75% of all ganglion cells in the retina,^18^ Prdm16+ cells account for just 2% of the total ganglion cell population.

**Figure 3:**
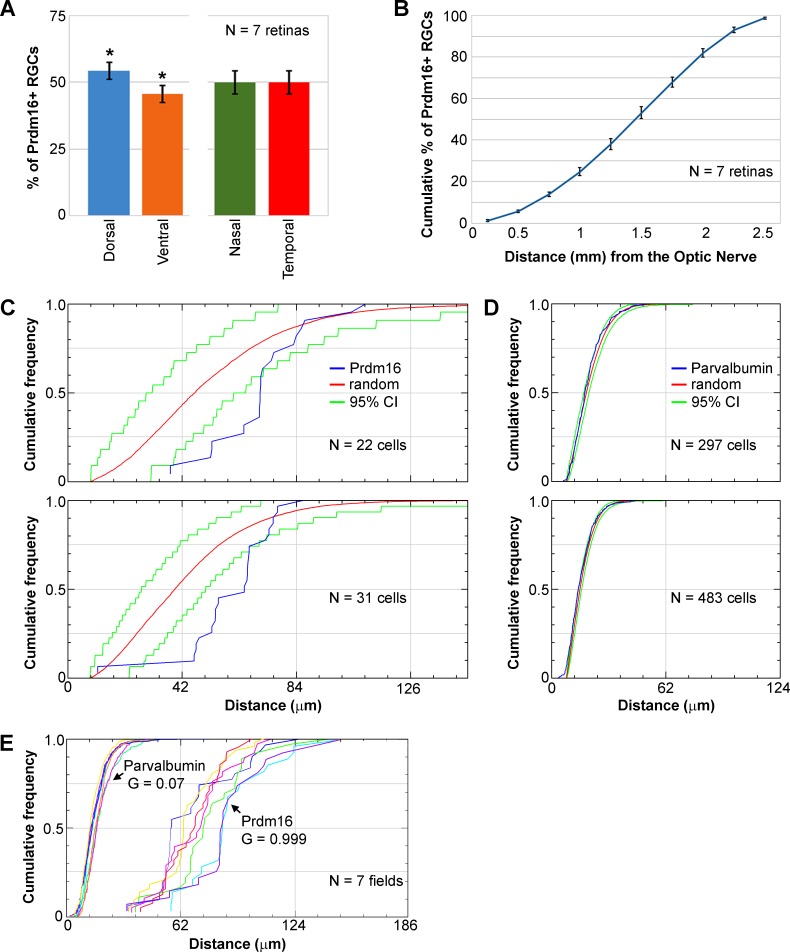
Prdm16+ ganglion cells are nonrandomly distributed. We excluded vascular cell nuclei from these analyses. (A) The distribution of Prdm16+ ganglion cells is equivalent between the nasal (green) and temporal (red) halves of the retina, but the dorsal (blue) retina has about 20% more Prdm16+ cells than the ventral (orange) half. * t-test, P < 0.001. Error bars denote the SD from quantifying seven whole mount retinas. (B) Cumulative distribution plot of Prdm16+ ganglion cells moving radially from the optic nerve (0 mm) to the periphery (2.5 mm). The distribution is linear (regression, r^2^ = 0.97), indicating that Prdm16+ ganglion cells are equally abundant from the central to peripheral retina. There are modestly fewer Prdm16+ ganglion cells near the optic nerve and beyond 2.25 mm. Error bars represent the SD from quantifying 7 retinas at 10 radial distances from the optic nerve. (C–E) Nearest neighbor analysis of Prdm16+ ganglion cells and parvalbumin+ somas shown as cumulative distribution plots. (C) Two examples of the nearest neighbor distribution of Prdm16+ cells (blue) in ×200 flatmount images compared to a random distribution (red) calculated using the same number of events. The green lines represent the 95% confidence interval (CI) of the random distribution. Note that the Prdm16 distributions are strongly right-shifted versus the random distribution and have a steeper slope. The distance between cells is shown on the abscissa (in μm). (D) Two examples of nearest neighbor distributions of heterogeneous parvalbumin+ soma within the ganglion cell layer. The blue lines showing the parvalbumin distribution closely parallel the red lines marking the random pattern. (E) Cumulative plot of Prdm16+ ganglion cell and parvalbumin+ soma nearest neighbor distances from seven costained (color matched) ×200 magnification fields. The average G-function spatial distribution index function values (0.0–1.0, random to uniform) indicate that parvalbumin+ soma are randomly distributed and Prdm16+ ganglion cells are uniformly distributed.

Their paucity and consistent parvalbumin and Brn3a coexpression suggest that Prdm16+ cells represent a single RGC subtype. Cells of the same subtype often repulse one another to tile their somas and dendrites evenly across the retina.^32,71^ If Prdm16 marks a single subclass of RGC, we predicted that their nuclei would be uniformly distributed in the GCL. In contrast, if Prdm16 marks multiple subtypes, a random distribution of nuclei would be expected. To evaluate this, we first measured the distribution of Prdm16+ ganglion cells across the retina using flatmount preparations. We quantified the number of Prdm16+ cells in the dorsal, ventral, nasal, and temporal regions of the retinas (Fig. 3A). Prdm16+ ganglion cells were equally distributed between the nasal and temporal halves of the retina, but showed a modest enrichment in the dorsal retina compared to the temporal region (54.4% ± 3.1% SD versus 45.6% ± 3.2% SD, *n* = 7, *t*-test, *P* < 0.001; Fig. 3A). Prdm16+ ganglion cells in these retinas were then quantified at increasing radial distances from the optic nerve and plotted as a cumulative distribution (Fig. 3B). The distribution was linear (regression, *r*^2^ = 0.97), indicating that Prdm16+ nuclei were equally distributed in the radial dimension (Fig. 3B). There were modestly fewer Prdm16+ ganglion cells close to the optic nerve and within the far peripheral retina (Fig. 3B). To further test whether Prdm16+ ganglion cells are uniformly distributed, we conducted nearest neighbor analysis using a 2D spatial statistics plug-in for ImageJ^66,67^ (Figs. 3C–E). The software generated cumulative nearest neighbor distribution plots, and simulated random distributions and 95% confidence intervals based on the number of cells in each image (Figs. 3C–E). The nearest neighbor distances of Prdm16+ nuclei are strongly right-shifted compared to the simulated random pattern and the slope of the distribution is more vertical (Fig. 3C). In general, few nuclei were close together or very far apart (Figs. 3C, 3E). We observed that the median nearest neighbor distance between Prdm16+ nuclei was 68.2 μm (±9.2 μm SD, *n* = 16 fields). Only 25% of nuclei were closer together than 59.7 μm (±9.3 μm SD) or farther apart than 79.8 μm (±8.4 μm SD). We next examined parvalbumin+ somas in the ganglion cell layer. This represents a complex group of cells that includes the Prdm16+ ganglion cell population. As expected for a heterogeneous population, the cumulative nearest neighbor distribution for parvalbumin+ somas closely mirrored the random pattern (Fig. 3D). We used the G-function spatial distribution index to test for random versus uniform distributions.^66,67^ An index close to 0.0 indicates a random distribution while an index near 1.0 is nonrandom. Parvalbumin+ somas had an average G index of 0.07 (±0.08 SD), consistent with a random distribution (Fig. 3E). The G index of Prdm16+ nuclei (0.999 ± 0.003 SD) was significantly greater (*t*-test, *P* < 0.0001) than for parvalbumin+ somas (Fig. 3E), indicating that Prdm16+ ganglion cells are uniformly distributed.

**Figure 4:**
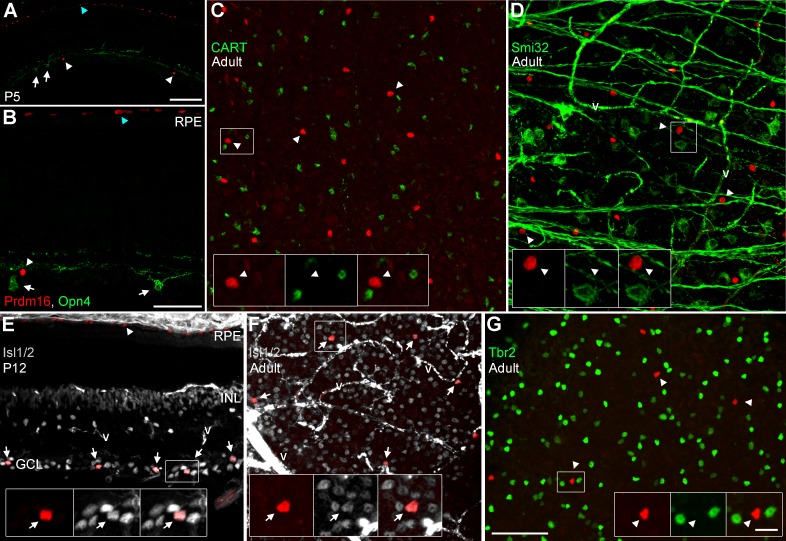
Prdm16+ ganglion cells express Isl1/2, but not other subtype-restricted markers. Sections and flatmounts immunostained with Prdm16 (red). (A, B) P5 retinal sections stained with antibodies against melanopsin (green; Opn4). Melanopsin+ RGCs (arrows) do not coexpress Prdm16 (arrowheads). Blue arrowheads mark the RPE. (C) Adult retinal flatmount stained for CART (green). Prdm16+ ganglion cells do not coexpress CART (arrowheads, insets). (D) Adult flatmount stained for nonphosphorylated neurofilament H (Smi32; green). Prdm16+ cells (arrowheads, insets) do not coexpress Smi32. The fibrous green signal orthogonal to the axons represents nonspecific staining of blood vessels (v). (E) P12 retinal section stained for Isl1/2 (gray). All Prdm16+ cells in the GCL coexpress Isl1/2 (arrows, insets). Arrowhead marks the RPE. (F) Adult flatmount retina stained for Isl1/2 (gray). All Prdm16+ cells coexpress Isl1/2 (arrows, insets). The intense signal in (E) and (F) represents nonspecific staining of vascular structures (v). (G) Adult flatmount stained for Tbr2. No Prdm16+ cells (arrowheads, insets) coexpress Tbr2. Scale bars: 100 μm for (A) and 50 μm for (B), (C) through (G). Scale bar for insets is 10 μm.

The uniform distribution pattern we observed suggested that Prdm16 marks a single ganglion cell subtype. To identify which subtype, we examined several additional RGC markers (Fig. 4; Table) and compared our results to the subtype classification scheme described by Völgyi and colleagues.^30^ While not comprehensive, their morphological scheme provides a good framework for comparison to other studies. We first costained sections for melanopsin (Opn4), which marks intrinsically photosensitive RGCs.^2^ There was no overlap with melanopsin (Figs. 4A–B), indicating that Prdm16 does not mark any of the Opn4+ intrinsically photosensitive ganglion cell subtypes. We compared our melanopsin, calretinin, Brn3a, and parvalbumin overlap data with the descriptions by Völgyi and colleagues^30^ to substantially narrow the list of possible ganglion cell subtypes to four candidates (G_1_, G_9_, G_15_, and G_16_; Table). To discriminate between these types we examined retinal flatmounts for CART, a marker of the G_16_ subtype, better known as ON-OFF directionally sensitive ganglion cells (ooDSGCs).^32,72^ No overlap of Prdm16 and CART was observed (Fig. 4C), thus Prdm16+ ganglion cells were not ooDSGCs. The nonphosphorylated form of neurofilament heavy chain is detected by the monoclonal antibody Smi32. This marks several RGC subtypes, including G_1_ cells.^34^ Prdm16 did not overlap with Smi32 (Fig. 4D), arguing that Prdm16 does not mark G_1_ ganglion cells. This left only the G_9_ and G_15_ (better known as direction sensitive J-RGCs)^73^ subtypes as candidates for the Prdm16 cohort of RGCs. Parvalbumin expression was not previously seen in G_15_ cells, strongly suggesting that Prdm16 marks only the G_9_ subclass of RGCs (Table). Parvalbumin levels were typically low in our Prdm16+ cells and its expression was previously reported to be heterogeneous.^70^ Thus, it is possible that parvalbumin expression does not fully distinguish between G_9_ and G_15_ subtypes. In an attempt to bypass this potential limitation, we examined two additional RGC markers. We first tested Isl1/2 expression, which marks a large cohort of RGCs that preferentially project to vision forming areas of the brain.^74^ We observed that all Prdm16+ ganglion cells coexpressed Isl1/2 (Figs. 4E–F). In contrast, we observed no Prdm16 overlap with Tbr2 (Fig. 4G), which typically marks RGCs that project to nonimage forming areas of the brain.^28^ Although these two markers have not been explicitly characterized in the G_9_ and G_15_ subtypes (Table), G_15_ cells have been shown to project their axons to vision forming areas^75^ and are likely to be Isl1/2+ and Tbr2-negative.

**Table:**
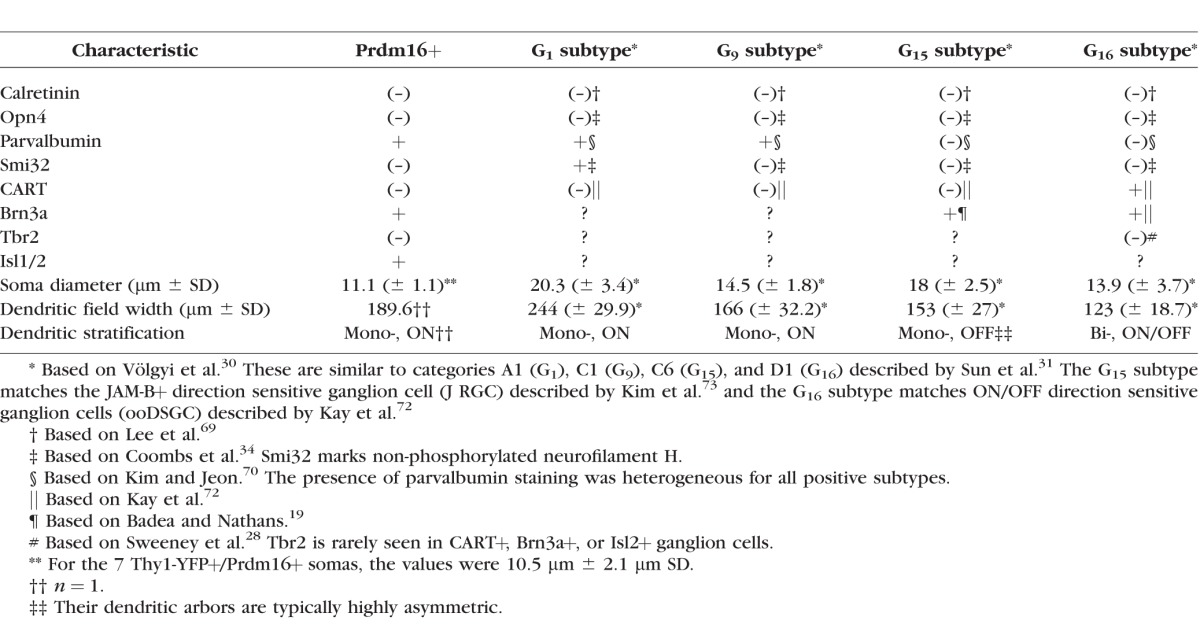
Ganglion Cell Subtype Characteristics

**Figure 5:**
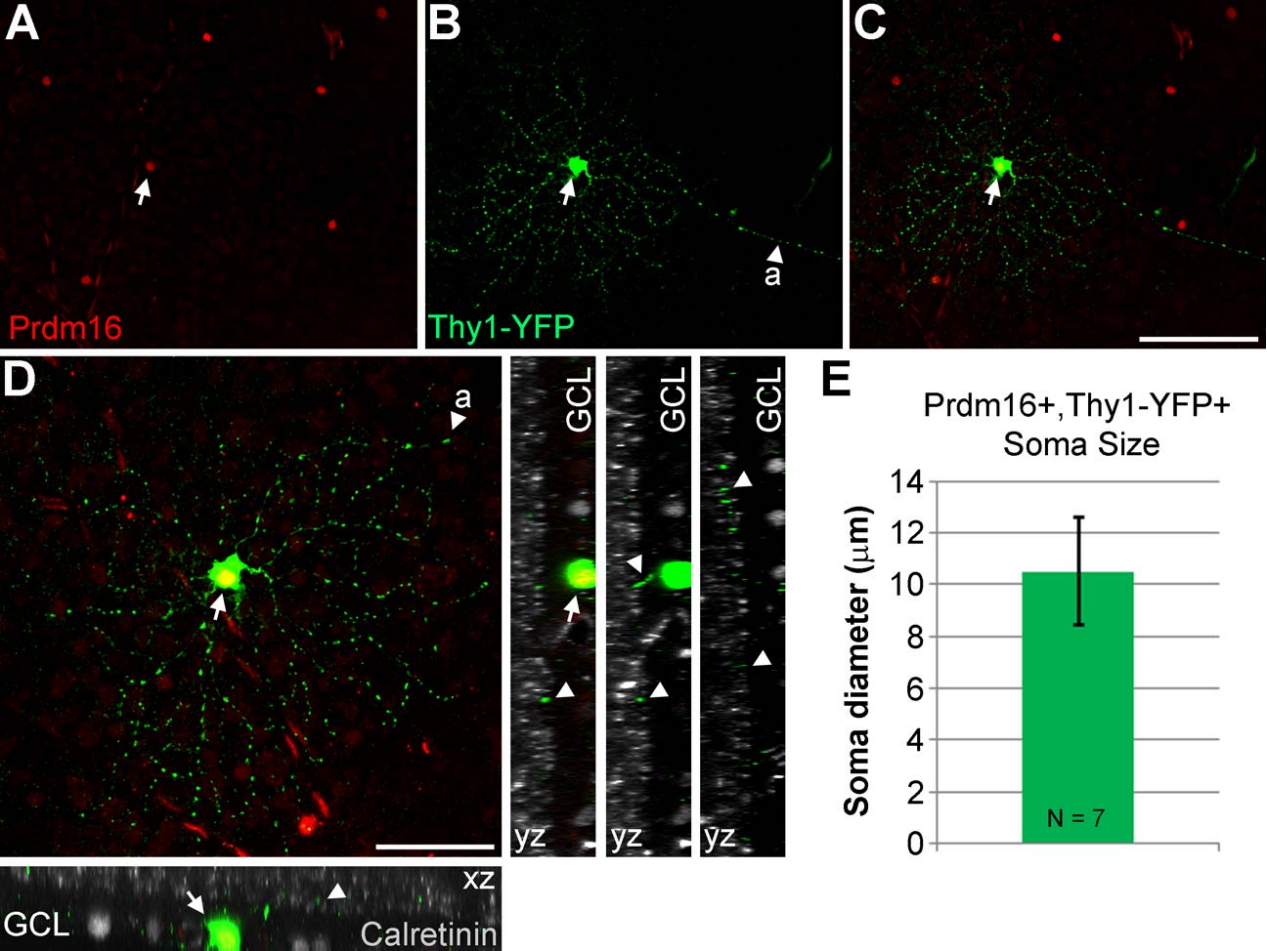
Morphologic characteristics of Prdm16+ ganglion cells. (A–D) A Thy1-YFP-H transgenic mouse retinal flatmount stained with antibodies to GFP (green); Prdm16 (red); and calretinin (gray). One round Prdm16+ nucleus coexpresses YFP (arrows) and its axon is conspicuous ([A], arrowheads). This cell is located about two-thirds of the way to the retinal periphery (left). (D) A maximum intensity projection image of “C,” rotated and magnified to highlight the dendritic arbor. The dendritic field was 189.6 μm in diameter and circular in shape with few dendrites crossing one another. Dendrites tend to branch sharply. XZ and YZ views of the cell with calretinin to mark the substrata of the inner plexiform layer. The dendrite staining (arrowheads) is localized between the inner and middle calretinin band, within the ON portion of the inner plexiform layer. Scale bars: 100 μm for (A) through (C), and 50 μm for (D). (E) Plot of the average soma diameter measured from seven Prdm16+/Thy1-YFP+ cells. The error bar represents the SD of the seven cell somas measured.

Since we were unable to unequivocally distinguish between G_9_ and G_15_ subtypes by marker expression, we compared the morphology of the cell soma and dendritic field (Table). To bypass the limitation of Prdm16 nuclear localization, we examined ganglion cells in *Thy1-YFP-H* transgenic mice.^62^ In these mice a very small number of RGCs per eye express cytoplasmic YFP, allowing for the characterization of morphologic features.^72^ The expression of YFP appears to be random, marking many (if not all) RGC subtypes. We costained retinas with antibodies to Prdm16, GFP (detects YFP), and calretinin to mark the synaptic strata of the IPL.^76^ Out of 107 retinas stained, we identified 7 Prdm16+/YFP+ ganglion cells (Fig. 5). This was significantly fewer overlapping cells than expected by chance when 2% of all RGCs express Prdm16. Assuming a rather conservative estimate of 1000 YFP+ RGCs screened, the probability of seeing ≤7 overlapping cells was 0.00071 (binomial distribution). This argues that Prdm16+/YFP+ RGCs are severely underrepresented in *Thy1-YFP-H* transgenic mice. Although we observed seven cells, only one of them had a complete dendritic arbor that we could also image in isolation (Figs. 5A–D). This RGC had a circular dendritic field with dendrites that tended not to cross one another. The field diameter was medium-sized (189.6 μm), and the dendrites were monostratified in the ON portion of the IPL (Fig. 5D; Table). We were able to measure the soma diameter of all 7 Prdm16+/YFP+ cells, which was small at 10.5 μm (±2.1 μm SD; Fig. 5E; Table) and consistent with the parvalbumin costains (11.14 μm ± 1.04 μm SD; Figs. 2E–I). This small soma size matched best with the G_9_, but not the significantly larger G_15_ RGC subtype (Table). Though limited, the dendritic morphology of the Prdm16+ cell is also consistent with the G_9_ subtype (Table). Taken together, the combination of immunostaining, distribution, and morphologic data strongly suggest that Prdm16 marks a single ganglion cell subtype that most resembles the G_9_ population described by Völgyi and colleagues.^30^

### Prdm16 Expression Marks Large Vascular Structures

**Figure 6:**
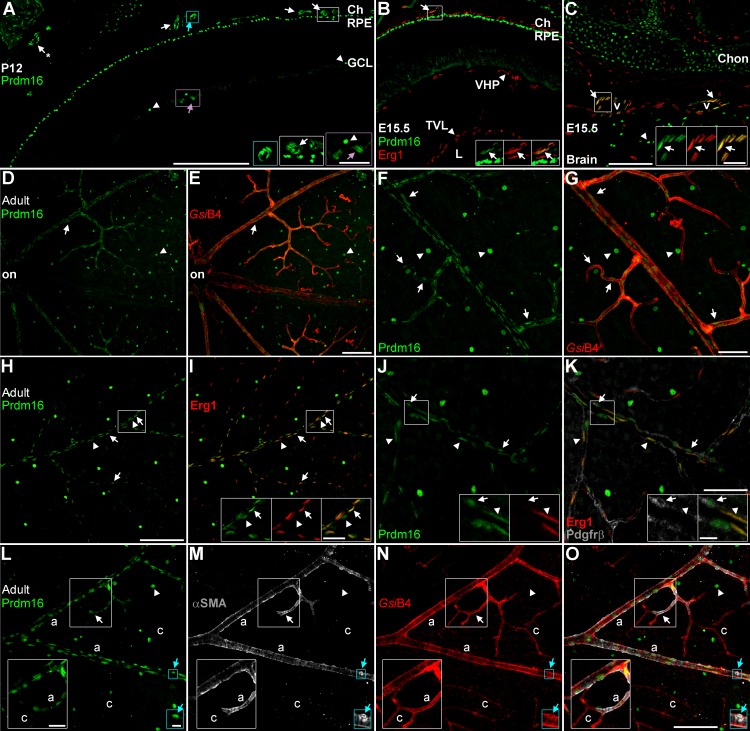
Prdm16 marks large diameter vessels inside and outside the eye. Sections and retinal flatmounts stained with Prdm16 (green) and vascular markers. (A) At P12, Prdm16 marks the RPE and a subset of ganglion cells (arrowheads). Elongated Prdm16 nuclei are seen in the choroid (ch) (blue, white arrows, insets); the retina (purple arrows, insets); and near extraocular muscles (*-arrow). These nuclei form tubular structures indicative of blood vessels. No Prdm16+ capillaries are seen within the retina and only a small subset of the choroidal vessels are labeled. (B, C) Horizontal sections of an E15.5 embryo stained for Erg1 (red), which marks vascular endothelial cell nuclei. In the developing eye (B), Prdm16 labels the RPE and a small subset of the choroidal vessels (arrows, inset). The fetal vascular networks (VHP, vasa hyaloidia propria; TVL, tunica vasculosa lentis) in the vitreous (arrowheads) did not coexpress Prdm16. Caudally, (C) large diameter vessels adjacent to the brain express Prdm16 and Erg1 (arrows, insets). Prdm16+ neural cells (arrowheads) and chondrocytes (Chon) are evident. (D–O) Adult retinal flatmounts. (D–G) Flatmount stained with GsiB4 lectin (red) to mark all blood vessels. Only a subset of large diameter vessels coexpress Prdm16 (arrows). Prdm16+ ganglion cells are marked with arrowheads. ON, optic nerve. (H, I) Flatmounts stained with Erg1 (red). Within positive vessels, essentially all Erg1+ endothelial cells coexpress Prdm16 (arrows, insets). (J, K) High power view of flatmounts stained with Erg1 (red) and Pdgfrβ (gray) to mark mural cell membrane. A subset of mural cells coexpresses Pdgfrβ and Prdm16 (arrows, insets). Arrowheads mark Prdm16+ endothelial cells. (L–O) Staining with GsiB4 lectin (red) and α-smooth muscle actin (αSMA; gray) to mark all vessels and arteries/arterioles (a), respectively. Prdm16 staining correlated tightly with αSMA staining (arrows, insets). Capillaries (c) did not express Prdm16 or αSMA. Some Prdm16+ cells are strongly αSMA+ (blue arrows, insets), suggesting that they are vascular smooth muscle cells. Arrowheads mark Prdm16+ ganglion cells. Scale bar: 250 μm for (A); 100 μm for (B) through (E), (H), (I), (L) through (O); and 50 μm for (F), (G), (J), (K). Scale bars for insets are 50 μm for (A); 25 μm for (B), (C), (H) (I), (L) through (O); and 10 μm for (J) and (K) and the blue insets in (L) through (O).

As noted above, we observed elongated Prdm16+ nuclei along the inner retinal surface (Fig. 2A). This pattern was seen with both anti-Prdm16 antibodies (data not shown), suggesting that Prdm16 also marks blood vessels. To confirm this, we costained sections and retinal flatmounts with Prdm16 and vascular markers (Fig. 6). At P12, we observed elongated Prdm16+ nuclei in the GCL, the choroid, and near extraocular muscles (Fig. 6A). These nuclei were often clustered into tube-like shapes, indicative of blood vessels (Fig. 6A). We then examined sections stained with Erg1, which marks endothelial cell nuclei.^77^ In E15.5 retinas, before Prdm16+ ganglion cells and retinal vessels are formed, we did not observe any Prdm16 staining in the retina or within the fetal vascular networks of the vitreous space (Fig. 6B). However, a subset of Erg1+ vascular endothelial cells within the developing choroid coexpressed Prdm16 (Fig. 6B). To determine whether Prdm16 marks endothelial cells outside the eye, we examined the same E15.5 horizontal sections in the developing brain region. In addition to the expected staining of chondrocytes and some neural cells,^55,57,59^ Prdm16 marked Erg1+ endothelial cells of several large diameter vessels adjacent to the brain (Fig. 6C). To better gauge retinal vessel staining, we examined Prdm16 staining in adult retinal flatmounts. Round Prdm16+ ganglion cell nuclei were evenly dispersed, while elongated nuclei overlapped with the *Griffonia simplicifolia* lectin (*Gsi*B4) that marks all blood vessels^78^ (Figs. 6D–G). While all elongated Prdm16+ nuclei overlapped with *Gsi*B4, only a subset of the *Gsi*B4+ vessels coexpressed Prdm16 (Figs. 6D–G). To determine which cell types within the vessels expressed Prdm16, we colabeled with Erg1 to mark endothelial nuclei and Pdgfrβ to mark mural cell membranes.^79,80^ Within vessels that expressed Prdm16, most (if not all) of the Erg1+ endothelial cells coexpressed Prdm16 (Figs. 6H–K). In these vessels, a subset of Pdgfrβ+ mural cells coexpressed Prdm16 (Figs. 6H–K). Since only a subset of vessels contained Prdm16+ cells, we tested whether this correlated with arterioles, venuoles, or capillaries. We costained flatmounts with *Gsi*B4 and α-smooth muscle actin (αSMA) to label all vessels and arterioles, respectively^81^ (Figs. 6L–O). We observed that Prdm16+ vessels were always ensheathed in αSMA+ vascular smooth muscle cells (vSMCs) characteristic of arterial vessels, indicating that Prdm16 marked only arterioles in the retina (Figs. 6L–O). The presence of Prdm16+ nuclei within a vessel abruptly stopped at the same location as αSMA, marking the boundary between arterioles and capillaries (insets, Figs. 6L–O). We also observed that some Prdm16+ nuclei were closely surrounded by αSMA staining, suggesting that Prdm16+ mural cells express αSMA and represent vSMCs (blue insets, Figs. 6L–O).

## Discussion

We examined the expression of Prdm16 in the developing and adult retina. In addition to its previously identified expression in the RPE,^57,61^ we found that Prdm16 was made by 2% of ganglion cells. The combination of morphology, markers, and distribution patterns suggests that Prdm16 marks a single RGC subtype. We also observed that large blood vessels expressed Prdm16, both in endothelial and mural cells. Whether Prdm16 controls the development of RGCs and blood vessels remains to be determined.

### Prdm16 Marks a Single Subtype of Ganglion Cells

Prdm16 was first expressed by ganglion cells at E16.5, days after the peak of RGC genesis in the mouse and before overt subtype morphologic features are present.^4–7,75^ These ganglion cells formed a relatively uniform distribution across the adult retina, suggesting that they represent a single subtype. Identifying which subtype expresses Prdm16 is made difficult by the nuclear localization of the protein and by the relatively small number of subtype-specific markers available. Using the Völygi classification scheme^30^ as a framework for comparison, Prdm16+ ganglion cells were limited to the G_1_, G_9_, G_15_, and G_16_ subtypes due to their lack of calretinin and melanopsin coexpression. Six more recently described RGC subtypes express either calretinin^39^ (F-mini ON, F-midi-ON, F-mini-OFF, F-midi-OFF) or CART^40^ (R, RDS). The absence of costaining with either of these markers indicates that Prdm16 does not mark these six ganglion cell subtypes. Furthermore, the lack of CART and Smi32 staining argues that Prdm16 does not mark the G_16_ and G_1_ subtypes, respectively. Prdm16+ cells are unlikely to be G_15_ (J) type ganglion cells because they coexpress parvalbumin. Taken together, the histologic data suggests that Prdm16 marks ganglion cells that closely resemble the G_9_ subtype. This assignment is supported by morphology data from the *Thy1-YFP-H* transgenic line. In particular, the small soma size and circular ON-laminated dendritic arbor was consistent with G_9_ cells, but not G_15_ cells that have large somas and OFF-laminated wedge-shaped dendritic arbors.^73^ It should be noted that several markers were characterized using RGC classification schemes that contained fewer ganglion cell subtypes,^34,69,70^ which were then converted to fit the Völygi scheme.^30^ There is thus some uncertainty about how well each marker fits a given subtype designation. It is possible that Prdm16 does not mark the G_9_ subtype, but rather defines another ganglion cell subtype with similar marker, distribution, and morphologic properties. It is also possible that Prdm16+ cells represent a family of closely related subtypes with modestly different physiological functions that have been grouped into the G_9_ category by Völygi and colleagues.^30^ Characterization of additional subtype markers and the generation of mice that express membrane-localized fluorescent proteins in the Prdm16 pattern will better define subtype identity.

The function of Prdm16+ ganglion cells is unknown, but some features of the G_9_ population have been postulated.^30^ G_9_ ganglion cells are likely ON-type as their dendrites are monostratified in the ON portion of the IPL. These cells are one of just a few subtypes not coupled by gap junctions to amacrine or other ganglion cells.^30^ RGCs expressing Isl1/2 and Brn3a typically project to image forming areas,^18,74,82,83^ whereas Tbr2+ cells project to nonimage forming areas of the brain.^28^ Since Prdm16+ ganglion cells express Isl1/2 and Brn3a, they may project to image forming areas. Evidence shows that Prdm16 affects the levels of reactive oxygen species and energy metabolism in neural precursors.^55,59^ This raises the possibility that Prdm16+ ganglion cells have different energetic needs compared to other ganglion cell subtypes. The creation of transgenic mice that specifically modify or ablate Prdm16+ ganglion cells will allow physiologic and behavioral tests of their function.

### The Role of Prdm16 in Ganglion Cell Development

The rodent retina contains more than 30 distinct ganglion cell subtypes. How this diversity is programmed during development is largely unknown. Prdm16 regulates cell type identity in hematopoietic stems cells and brown adipose.^41,53–56,58^ Prior work correlating cell cycle exit to the genesis of ganglion cell subtypes suggests that they are not formed in a specific sequence.^84^ Thus, the relatively late onset of Prdm16 expression may indicate a role in subtype formation after the commitment to ganglion cell fate has occurred. In this model, Prdm16 may directly instruct subtype development within a pool of otherwise uncommitted ganglion cells. Since Prdm16+ ganglion cells are rare and evenly distributed, cell-cell feedback mechanisms could activate Prdm16 and establish subtype identity. Alternatively, Prdm16 may act downstream of any fate choice decision, regulating key subtype-specific physiologic and morphologic features. Discriminating between these models will require *Prdm16* gain- and loss-of-function studies. Loss-of-function studies are complicated by the observation that *Prdm16* mutants die around birth, days before ganglion cell subtypes can be readily measured.^53^ Moreover, retinal architecture was disrupted in a subset of *Prdm16* mutants around birth, suggesting that *Prdm16* function in the RPE and/or nearby cell types is necessary for normal retinal development.^57^ Overcoming these barriers will require the deletion of *Prdm16* specifically from the developing retina.

It has been proposed that more broadly expressed transcription factors intersect to specify ganglion cell subtype choice.^23,32,39^ These types of combinatorial mechanisms likely operate upstream of *Prdm16* since it is expressed by a single ganglion cell subtype. Prime candidates to regulate *Prdm16* are Brn3a and Isl1/2. However, the intersection of broadly expressed Brn3a and Isl1/2 is expected to occur in many more ganglion cells than express Prdm16.^23^ This argues that additional transcription factors or signaling cascades intersect to regulate *Prdm16* expression or function. Prdm16 can interact with Smad proteins, potentially affecting TGFβ and Bmp signaling cascades in subsets of ganglion cells to control subtype identity.^41,57,85–88^ The structure of *Prdm3* (*Evi1*, *Mecom*) is closely related to *Prdm16* and these genes are coexpressed in several domains, including hematopoietic stem cells and craniofacial structures, where they act similarly to control development.^41,42,89–91^ *Prdm3* mRNA is also seen in the developing retina,^52,92^ but it is unknown whether Prdm3 cooperates with Prdm16 or other transcription factors to regulate ganglion cell subtype formation. Discovering the factors upstream and downstream of *Prdm16* expression will help uncover the mechanisms that regulate ganglion cell subtype fate choice.

### Prdm16 is Expressed by Large Vascular Structures

While Prdm16 expression has been observed in hematopoietic stem cells and the developing heart,^41,55,57,58,61^ we found no reports of its expression in developing or mature blood vessels. Prdm16 expression was observed in endothelial cells and some mural cells of large diameter vessels, but not small diameter capillaries. We observed Prdm16 staining of vascular structures in the retina and choroid, but also adjacent to the extraocular muscles and within the developing head. Thus, Prdm16 expression is not limited to vascular structures of the eye. In the retina, Prdm16+ vessels were surrounded by α-SMA+ smooth muscle cells, suggesting that it labels arteries and arterioles. While the fetal vasculature of the eye contains arterioles,^93^ we did not observe Prdm16 staining of these vessels. This may be due to the small size of the vessels, maturation status, or their transient nature.

Whether Prdm16 regulates vascular development is unknown. The closely related Prdm3 transcription factor has been shown to cooperate with Prdm16 in fish craniofacial development and has similar functions in mammalian hematopoietic stem cells.^41,58,89–91^ Of note, *Prdm3* mutant mice die around E15.5, apparently due to vascular defects.^89^ Our data and those on Prdm3 are consistent with a regulatory role for Prdm16 during vascular development. Since *Prdm16* null mice die later in development than *Prdm3* mutants, any role *Prdm16* plays in vascular development: (1) is compensated for by the action of other factors like Prdm3, (2) occurs late in gestation, (3) or occurs only in a subset of vessels. The role of Prdm16 in mature vessels is also unknown. Single nucleotide polymorphisms in *PRDM16* have been associated with migraine headache,^94–100^ raising the possibility that *PRDM16* affects vascular function in the adult brain.
